# Weak Hand Grip Strength Is Associated with Alexithymia in Outpatients in a Mexican Population

**DOI:** 10.3390/brainsci12050576

**Published:** 2022-04-29

**Authors:** Alma Delia Genis-Mendoza, Ana Fresán, Thelma Beatriz González-Castro, Sherezada Pool-García, Carlos Alfonso Tovilla-Zárate, Rosa Giannina Castillo-Avila, Pedro Iván Arias-Vázquez, María Lilia López-Narváez, Humberto Nicolini

**Affiliations:** 1 Laboratorio de Genómica de Enfermedades Psiquiátricas y Neurodegenerativas, Instituto Nacional de Medicina Genómica, Ciudad de México 14610, Mexico; adgenis@inmegen.gob.mx; 2 Subdirección de Investigaciones Clínicas, Instituto Nacional de Psiquiatría Ramón de la Fuente Muñíz, Ciudad de México 14370, Mexico; fresan@imp.edu.mx; 3 División Académica Multidisciplinaria de Jalpa de Méndez, Universidad Juárez Autónoma de Tabasco, Jalpa de Méndez 86205, Mexico; thelma.glez.castro@gmail.com; 4 Hospital General de Comalcalco “Dr. Desiderio G Rosado Carbajal”, Secretaría de Salud, Comalcalco 86300, Mexico; shepoga70@hotmail.com; 5 División Académica Multidisciplinaria de Comalcalco, Universidad Juárez Autónoma de Tabasco, Comalcalco 86300, Mexico; pivanav@gmail.com; 6 División Académica de Ciencias de la Salud, Universidad Juárez Autónoma de Tabasco, Villahermosa 86100, Mexico; gianninaavila2012@hotmail.com; 7 Hospital Chiapas Nos Une Dr. Gilberto Gómez Maza, Secretaría de Salud de Chiapas, Tuxtla Gutiérrez 29045, Mexico; dralilialonar@yahoo.com.mx

**Keywords:** alexithymia, hand grip strength, weak hand grip strength, TAS-20

## Abstract

Hand grip strength has been considered as a possible marker for metabolic and psychiatric disease. To date, however, no research has focused on the association between alexithymia and hand grip strength. The objective of the present study was to investigate the correct association between hand grip strength and alexithymia. A cross-sectional study was carried out in Comalcalco, Tabasco, México. A total of 246 individuals were included. Hand grip strength was evaluated in the dominant hand using a Takei^®^ portable digital dynamometer. Alexithymia was measured using the Toronto Alexithymia Scale (TAS-20). Two linear regression models adjusted by confounders were used to determine the association between alexithymia and hand grip strength. The rate for positive alexithymia was 39.0% (n = 94). Individuals with alexithymia showed a weaker hand grip strength than the comparison group (t = 2.4, 244 df, *p* = 0.01). Individuals with alexithymia had significantly reduced levels of hand grip strength (β = −0.39 ± 0.14; *p* = 0.006); after additional adjustment for clinical variables, decreased hand grip strength remained (β = 8.00 ± 1.86; *p* ≤ 0.001). Our results suggest that a decrease in hand grip strength could be associated with alexithymia. This measurement could be useful as a predictive marker for the identification of alexithymia in Mexican individuals who attend outpatient clinics.

## 1. Introduction

Alexithymia is an emotional dysregulation defined as the inability to perceive and express internal emotions treated as a stable personality trait, which, along with other personality factors, predisposes one to the presence of a variety of mental and physical diseases [1,2]. Alexithymia is considered a personality trait in which the following interrelated components are involved: (a) difficulty identifying one’s own feelings (DIF), (b) difficulty describing feelings (DDF), and (c) externally oriented thinking (EOT) [3]. To date, there is broad agreement among alexithymia theorists that DIR, DDF, EOT, and difficulty in fantasizing are the core components of the alexithymia construct [4]. This pattern has consistently been replicated across several self-reported measures, such as the 20-item Toronto Alexithymia Scale (TAS-20) [5,6].

As a personality trait, alexithymia can occur in conjunction with various physical and psychopathological disorders with a temporary role linked to them [7], such as depression, aggression, substance use, and suicidal ideation. Therefore, it is important to find indicators that facilitate its diagnosis and that can be used as predictive markers to avoid health complications.

In addition, studies have described an association between the intensity of pain and the degree of alexithymia [8,9], so it is relevant to consider this as a study variable.

On the other hand, the assessment of muscle strength has become an important marker of health and function in individuals with the presence of various diseases [10,11,12,13]. In this sense, hand grip strength (HGS) measured by a dynamometer is very useful as it is easy to perform measurements and is of low cost [14]. It has been used for diagnostic and prognostic purposes as a health marker/morbidity and physical function/disability predictor, and even as a survival/mortality predictor. Likewise, it has also been used in healthy individuals [15,16,17] and in individuals with various diseases such as diabetes [18], cardiovascular disease [19], kidney diseases [20], cancer [21], and HIV/AIDS [22] among others. 

Reports in older adults indicate that low levels of HGS are associated with deterioration in various areas of cognitive function [23,24], as well as with an increased risk of mental health disorders [25]. In particular, low levels of HGS have been associated with an increased risk of depression in older adults [26,27], with a higher prevalence in females [28]. 

Additionally, structural changes have been described in patients with depression, in which increased HGS has been associated with increased left and right hippocampal volume and reduced levels of white matter hyperintensity [29]. On the other hand, high levels of HGS have been reported as a factor associated with a lower prevalence of generalized anxiety in older adults [30].

Due to the described associations, the aim of the present study was to investigate the correlation between HGS and alexithymia, considering demographic (gender and age) and health features (tobacco and alcohol consumption, body mass index, reported medical condition, and the presence of pain) as confounders.

## 2. Materials and Methods

The present study was a cross-sectional correlational study performed with a convenience sampling method with individuals who were available and willing to participate. The study was conducted in Comalcalco, in Tabasco, Mexico, from September to December 2019.

### 2.1. Participants 

All the participants gave their verbal and written informed consent after the aim and procedures of the study were fully explained to them. The participants were recruited from the gynecology, traumatology, and internal medicine outpatient services of the General Hospital of Comalcalco “Dr. Desiderio G Rosado Carbajal”. The Carbajal hospital is categorized as a second-level care center; therefore, it only has five areas of specialization (gynecology, traumatology, internal medicine, pediatrics, and surgery). However, pediatric services (<18 years of age) and surgery (subjects scheduled for or having recently undergone surgical procedures) were excluded from this study. 

Those aged at least 18 years old and able to read and write (or have a companion that could help them with these tasks), with no history of mental health diagnoses or evident cognitive impairment at the time of the study, were included. Participants with physical limitations in their dominant hand were not included in the study as their hand grip strength assessment could be biased. For instance, patients with a history or current diagnosis of hand trauma, nerve injury, or carpal tunnel syndrome were excluded because it has been noted that the grip strength of the hand decreases in these types of conditions [31,32].

### 2.2. Measures

The demographic and current clinical data were gathered from a face-to-face interview with one of the researchers. Gender, age, marital status, current occupation, and years of education were collected as main the demographic features, while diagnoses for attendance (medical condition), presence of pain, body mass index (BMI), and current alcohol and tobacco consumption were the clinical variables registered from the interview. The BMI calculation (kg/m^2^) was determined according to the obesity task force criteria; body weight (kg) and height (cm) were collected as previously reported [33].

Hand grip strength (kg) was determined 3 times using a dominant hand trough dynamometer (Takei^®^; Scientific Instruments Co., Ltd., Tokyo, Japan). HGS was determined in the dominant hand using a Takei^®^ portable digital dynamometer (Scientific Instruments Co., Ltd., Tokyo, Japan), using the highest record of three attempts expressed in kilograms. The opening of the dynamometer handle was adjusted to the size of the participant’s hand and the measurements were taken with the participant in a standing position, with arms parallel to the body but not in contact with it, with the shoulder adduced, and a neutral forearm. HGS was determined 3 times using a dominant hand trough dynamometer (Takei^®^; Scientific Instruments Co., Ltd., Tokyo, Japan).

Alexithymia was measured using the 20 self-reported item Toronto Alexithymia Scale (TAS-20) [34,35]. This scale is widely considered the most valid and reliable instrument (total score internal consistency ≥ 0.80) for the measurement of alexithymia [36,37] and is divided into 3 factors: (a) difficulty identifying feelings (7 items), (b) difficulty describing feelings (5 items), and (c) externally oriented thinking (8 items) [38]. Each item is scored on a 5-point Likert agreement scale ranging from *strongly disagree* to *strongly agree*. The total score of the TAS-20 ranges between 20 and 200 points, which are obtained from the sum of each item, with higher scores reflecting greater alexithymia [36,39,40]. For the present study, we used a cutoff score higher than 61 in the total TAS-20 score to identify subjects with possible or definite alexithymia [39,40].

Pain was measured using a Visual Analogue Scale for Pain Assessment (VAS-P) as previously reported elsewhere [41,42]. Individuals with a VAS-P of 1 or more were considered patients with current pain.

### 2.3. Statistical Analysis

The analyses were conducted using IBM SPSS statistics v.21. We used descriptive statistics (means, percentages, frequencies, and ranges) to summarize the sample demographic and clinical features, and two linear regression models were performed to determine the association of alexithymia and HGS. The first model was adjusted for sex and age (both considered international benchmarks for HGS), while the second model was adjusted by the previous demographic features and clinical features (alcohol and tobacco consumption; reported medical condition, known/unknown due to the number of diagnoses reported; and presence of pain and BMI). Unstandardized regression coefficients (β) reflect the change in alexithymia values associated with a 1 kg increase in HGS. All the analyses were deemed significant with a *p*-value ≤ 0.05.

## 3. Results

### 3.1. Demographic and Clinical Characteristics

A total of 246 individuals were included in this study. The mean age of the participants was 40.2 ± 13.6 years; 88.6% (n = 218) were women and 11.4% (n = 28) were men. The majority of the participants were married at the time of the study (75.5%, n = 185), dedicated to household activities (77.2%, n = 190), and had 7.9 ± 4.0 years of education. 

Only 57.5% (n = 147) of the participants provided information on their medical condition for attending the outpatient clinic. The remaining 42.5% of participants did not report their current diagnosis because they were newly admitted patients and were pending the results of the studies carried out and the diagnosis was still in process. 

Cardiovascular diseases were the most reported (38.0%, n = 54) in those who identified their medical condition. Metabolic and gynecology–obstetric diseases occupied the second most common cause of medical consultation, both accounting for 33.1% (n = 47).

On the other hand, 14.6% (n = 36) and 4.5% (n = 11) of the participants indicated that they were current alcohol and tobacco consumers, respectively. The mean BMI was 29.3 ± 5.6. The results indicated the parameters of overweight and obesity among the study subjects. Finally, more than half of the sample reported currently having pain (61.4%, n = 151). Table 1 shows the demographic and clinical characteristics of the study population.

### 3.2. Association of Hand Grip Strength and Alexithymia

The mean TAS-20 score was 47.6 ± 14.5 [range 20–80], where 38.2% (n = 94) were considered to have possible/definite alexithymia. The mean combined hand grip strength in the overall sample was 22.0 ± 7.2 [range 2–47.9] kg. Patients with alexithymia exhibited a diminished HGS (mean = 20.6 ± 7.0) when compared to the group without alexithymia (22.9 ± 7.2; t = 2.4, df 244, *p* = 0.01). In the first linear regression model adjusted by sex and age, there was a negative association between HGS and alexithymia, where individuals with alexithymia had significantly reduced levels of HGS (β = −0.39 ± 0.14; *p* = 0.006). This association remained significant after additional adjustments for clinical variables (β = −0.37 ± 0.14; *p* = 0.008) with the presence of pain as an additional variable associated with alexithymia (see Table 2).

## 4. Discussion

Hand grip strength is the effort used to grasp an object and to perform different functional activities in daily life [43]. In this regard, HGS has been considered an important measure of intrinsic capacity, a vital sign of health, and a relevant biomarker of aging, to name a few examples [44]. This measurement has been of particular interest to many scientists and clinicians around the world due to its ease of use and low cost. There are reports on multiple diseases [45] and disabilities [46] where a decrease in HGS has been associated with negative repercussions on health. Likewise, the relationship between HGS and mental health has been studied, among which depression and suicidal behavior stand out [47]. However, despite the scientific evidence and the benefits of using this measurement, there are no previous studies that specifically address alexithymia. Therefore, in the present research, we evaluated the association between HGS and alexithymia, taking into account demographic (sex and age) and health feature (tobacco and alcohol consumption, body mass index, and the presence of pain) confounders. 

First, we measured the participants’ HGS. As expected, we found that it was weaker in individuals with alexithymia. These results support what has been previously described in various populations with psychiatric disorders [48,49]. For instance, a study described that a weaker HGS is longitudinally and transversely associated with depressive symptoms [50]. In this regard, a study in adult Korean population showed that every 1 kg increase in HGS decreased the probability of suicidal ideation in men and women, regardless of the depressive mood of the individuals [51]. The evidence found in previous reports and the results of our study suggest that a decrease in HGS could be directly or indirectly associated with some mental illnesses or their clinical manifestations, including alexithymia [25,52].

Furthermore, various factors that influence HGS have been identified, including age, gender, and pain [53]. Our findings showed that HGS was independently associated with alexithymia, where a decrease in HGS could be used as a predictive marker for its expression and severity. In this way, this measurement could be used in clinical settings and could be of great utility for identifying alexithymia, regardless of the gender and age of the patients. Even though there are no previous studies evaluating the relation between alexithymia and HGS, our results are consistent with previous associations that used this measurement in individuals affected with mental health disorders [54], where a weak HGS and alexithymia could exacerbate each other bi-directionally. 

Additionally, we performed a second model adjusted for demographic and clinical characteristics (age, gender, reported medical condition, presence of pain, alcohol consumption, tobacco consumption, and BMI). Our results suggest that pain could be indicative of the presence of alexithymia, which is consistent with previous studies that showed an association between pain and alexithymia. In a study carried out in a Japanese population, it was reported that the intensity of pain in the participants significantly increased the degree of alexithymia [8]. Another study conducted by Aaron et al. that compared participants with and without chronic pain found that individuals with pain had higher total scores of alexithymia and a greater difficulty in identifying feelings [9].

It is important to take into account that the present study has some limitations. The cross-sectional design limits the ability to make inferences about the causality between HGS and alexithymia, for which longitudinal studies are required. Due to the fact that the recruitment of patients was consecutive, in this study, the influx of female patients was greater. Additionally, we did not enquire if the presence of alexithymia in our sample was due to a traumatic experience in childhood, post-traumatic stress disorder, substance use disorders, or established structural damage. In this regard, depending on the type of alexithymia (including the degree and the etiology), the decrease in HGS could be related to changes in the structural volumes of the brain in specific or relief areas implicit in motor skills, as has previously reported [55,56]. Some hypothetical explanations could be cited to explain the probable mechanism underlying the link between HSG and alexithymia, for example, a neurotrophic theory. It is well known that contracting skeletal muscle is a source of neurotrophic factors that regulate brain synapses [51]. In this sense, a reduction in brain-derived neurotrophic factors has been associated with the genesis of common mental health-related traits [57,58]. Another potential mechanism could be inflammation and oxidative stress. It has been reported that a chronic state of inflammation is related to a decline in physical strength and mental health [51,59]. Additionally, lifestyle could be implicated in the relationship between HGS and alexithymia. For example, low vitamin D levels are related to an increase in inflammatory cytokine production, which has been identified as a common mechanism that may explain the complex association of mental health issues and risky health behavior, among other chronic diseases [51].

Our results should also be taken with caution as our population was attending medical services and may have active symptoms of their medical illness, and alexithymia may also be affected.

To the best of our knowledge, this is the first study to identify HGS as a measure related to alexithymia in a Mexican population. Our findings could contribute to the use of HGS measurements as an objective, easy-to-use, low-cost, and clinically useful tool in the routine evaluation of individuals with probable alexithymia. Our findings also provide possible long-term lines of research. In this sense, it is necessary to contemplate future longitudinal studies that further explore the causal processes between alexithymia and the reduction in HGS. 

## 5. Conclusions

Our results suggest that Mexican individuals who attend medical consultations in outpatient clinics could have alexithymia, which can be associated with HGS, as observed in our results. Therefore, the identification of a decrease in this parameter could be useful for patients to be evaluated in depth by a multidisciplinary team that includes a specialist in psychiatry for the assessment of alexithymia and to promote early diagnosis and timely treatment with the aim of improving the patient’s quality of life and avoiding the negative effect of alexithymia in the course and prognosis of the patients’ medical disease. Finally, it is necessary to carry out future studies with more appropriate designs and to replicate them in other populations to corroborate these results.

## Figures and Tables

**Table 1:** Demographic and clinical characteristics of the sample.

Demographic Characteristics	Total Sample (n = 246)
Gender (n, %)	
Men	28 (11.4)
Women	218 (88.6)
Age, years (mean, SD) [range]	40.2 ± 13.6 [18–80]
Years of education, years (mean, SD) [range]	7.9 ± 4.0 [0–19]
Marital status (n, %)	
Married	186 (75.6)
Single	40 (16.3)
Widow	8 (3.3)
Divorced	12 (4.9)
Current activity (n, %)	
Household related	190 (77.2)
Remunerated employment	43 (17.5)
Unemployed	9 (3.7)
Student	4 (1.6)
*Clinical characteristics*	
Reported medical condition * (n, %)	
Cardiovascular	54 (38.0)
Metabolic	47 (33.1)
Gyneco-obstetrics	47 (33.1)
Infectious	4 (2.8)
Respiratory	8 (3.3)
Other chronic disease	23 (16.2)
Tobacco consumption—yes (n, %)	11 (4.5)
Alcohol consumption—yes (n, %)	36 (14.6)
Current pain—yes (n, %)	151 (61.4)
Body mass index (BMI) (mean, SD) [range]	29.3 ± 5.6 [18.5–50.2]

* n = 142, from those who identified the medical reason for their attendance.

**Table 2:** Association of hand grip strength and alexithymia—regression models.

Characteristics	β Coefficient	S.E.	95% C.I. β	Significance
*Model 1: Regression model adjusted by gender and age*				
Age	−0.05	0.07	−0.19–0.09	0.46
Gender	−2.63	3.26	−9.05–3.78	0.42
**Hand grip strength**	**−0.39**	**0.14**	**−0.67–−0.11**	**0.006**
*Model 2: Regression model adjusted by demographic and clinical variables*				
Age	−0.05	0.07	−0.21–0.09	0.48
Gender	−3.56	3.17	−9.82–2.69	0.26
**Presence of pain**	**8.59**	**1.90**	**4.84–12.34**	**<0.001**
Alcohol consumption	−3.47	2.78	−8.95–2.00	0.21
Tobacco consumption	−3.64	4.86	−13.22–5.93	0.45
BMI	0.02	0.15	−0.28–0.34	0.87
Reported medical condition	−3.09	2.08	−7.19–1.00	0.13
**Hand grip strength**	**−0.37**	**0.14**	**−0.65–−0.10**	**0.008**

## Data Availability

Not applicable.
